# Deep convolutional neural networks for multiplanar lung nodule detection: Improvement in small nodule identification

**DOI:** 10.1002/mp.14648

**Published:** 2020-12-30

**Authors:** Sunyi Zheng, Ludo J. Cornelissen, Xiaonan Cui, Xueping Jing, Raymond N. J. Veldhuis, Matthijs Oudkerk, Peter M. A. van Ooijen

**Affiliations:** ^1^ Department of Radiation Oncology University Medical Center Groningen University of Groningen 9713 AV Groningen The Netherlands; ^2^ Department of Radiology Tianjin Medical University Cancer Institute and Hospital National Clinical Research Centre of Cancer 300060 Tianjin China; ^3^ Faculty of Electrical Engineering University of Twente 7500 AE Enschede The Netherlands; ^4^ Faculty of Medical Science University of Groningen 9713 AV Groningen The Netherlands

**Keywords:** computer‐aided detection, computed tomography, convolutional neural network, deep learning, pulmonary nodule detection

## Abstract

**Purpose:**

Early detection of lung cancer is of importance since it can increase patients’ chances of survival. To detect nodules accurately during screening, radiologists would commonly take the axial, coronal, and sagittal planes into account, rather than solely the axial plane in clinical evaluation. Inspired by clinical work, the paper aims to develop an accurate deep learning framework for nodule detection by a combination of multiple planes.

**Methods:**

The nodule detection system is designed in two stages, multiplanar nodule candidate detection, multiscale false positive (FP) reduction. At the first stage, a deeply supervised encoder–decoder network is trained by axial, coronal, and sagittal slices for the candidate detection task. All possible nodule candidates from the three different planes are merged. To further refine results, a three‐dimensional multiscale dense convolutional neural network that extracts multiscale contextual information is applied to remove non‐nodules. In the public LIDC‐IDRI dataset, 888 computed tomography scans with 1186 nodules accepted by at least three of four radiologists are selected to train and evaluate our proposed system via a tenfold cross‐validation scheme. The free‐response receiver operating characteristic curve is used for performance assessment.

**Results:**

The proposed system achieves a sensitivity of 94.2% with 1.0 FP/scan and a sensitivity of 96.0% with 2.0 FPs/scan. Although it is difficult to detect small nodules (i.e., <6 mm), our designed CAD system reaches a sensitivity of 93.4% (95.0%) of these small nodules at an overall FP rate of 1.0 (2.0) FPs/scan. At the nodule candidate detection stage, results show that the system with a multiplanar method is capable to detect more nodules compared to using a single plane.

**Conclusion:**

Our approach achieves good performance not only for small nodules but also for large lesions on this dataset. This demonstrates the effectiveness of our developed CAD system for lung nodule detection.

## 1 introduction

Lung cancer is one of the most malignant cancers, and is a leading cause of death among both men and women.^1^, ^2^, ^3^ It has been predicted that around 25% of all cancer deaths in the United States in 2019 are due to lung cancer.^4^ Early detection of lung cancer can give better treatment alternatives to patients and increase their survival chances.^5^ To improve early diagnosis, lung cancer screening trials, such as the National Lung Screening Trial (NLST),^6^ and the Dutch‐Belgian Randomized Lung Cancer Screening Trial (NELSON),^7^ have been implemented.

Although the implementation of lung cancer screening reduces the mortality rate of patients, it results in a heavy workload for radiologists. Computer‐aided detection (CAD) systems could play an essential role in assisting radiologists to find nodules efficiently. A CAD system generally consists of two stages: Suspicious candidate detection and false positive (FP) reduction. The aim of any CAD system for lung nodule detection is to reach a high sensitivity with a low FP rate. However, CAD systems still have not been widely used in clinical practice for various reasons, including lack of reimbursement and low sensitivity or high FP rates of the available systems.^8^, ^9^ The challenges of this task are mainly the large variety in nodule morphology and the detection of small nodules, which are easily missed.

With the development of artificial intelligence algorithms and the abundance of computational power, a large number of deep learning techniques have been successfully used in image processing fields. For example, Ronneberger et al. proposed the U‐net algorithm for biomedical image segmentation,^10^ which showed good performance in the IEEE International Symposium on Biomedical Imaging (ISBI) cell tracking challenge. The U‐net algorithm is widely used for segmentation tasks throughout the literature ever since.^11^, ^12^, ^13^, ^14^ Variations on this architecture were soon proposed, such as the improved model U‐net++ from Zhou et al.,^15^ which modifies the skip connections between encoder and decoder pathways in the network. This should reduce the semantic gap between feature maps from the decoder and encoder paths, which makes training more efficient. Considering network architectures for image classification, Tan et al. demonstrated that by scaling depth, width, and resolution,^16^ Efficient‐Net becomes more accurate for object classification assessed on the CIFAR‐10 dataset. Inspired by dense convolution networks,^17^ Huang et al. developed a more effective architecture for image classification by adding multiscale blocks.^18^

Meanwhile, various authors have reported automatic lung nodule detection algorithms using deep learning.^19^ In the effort to minimize false negatives and FPs, Wang et al. proposed a nodule‐size‐adaptive model that can measure the nodule sizes, types, and locations from three‐dimensional (3D) images.^20^ Moreover, Dou et al. used 3D convolutional neural networks to extract multilevel contextual information to reduce FPs,^21^ while Xie et al. utilized two‐dimensional (2D) convolutional neural networks for FP reduction.^22^ Another approach by Setio et al. combined the predictions from seven independent nodule detection systems and five FP reduction systems.^23^ Some of the detection systems were developed for specific types of nodules. In addition, Ozdemir et al.^24^ and Gruetzemacher et al.^25^ developed end‐to‐end systems for nodule detection by utilizing 3D convolutional neural networks based on V‐net and U‐net, respectively. Huang et al.^26^ proposed amalgamated‐convolutional neural networks with the input in three scales to detect nodules. Furthermore, Zhang et al. applied constrained multiscale Laplacian of Gaussian filters to localize potential nodule candidates and a densely dilated 3D convolutional neural network to reduce FPs.^27^ Besides, a vector quantization algorithm was used by Tan et al.^28^ to detect potential nodules and knowledge of shape, texture was infused to the FP reduction model. Additionally, to develop an efficient nodule detection system, a multicenter study with 39 014 cases was conducted by Cui et al.^29^ deep learning techniques.

In our previous work, we followed one of the clinical procedures: Maximum intensity projection. With projected images as input, convolutional neural networks (CNNs) were employed to identify nodule candidates.^30^ Nodule cubes with various sizes were extracted for reduction of FPs. The results showed that using maximum intensity projection can improve the performance of deep learning‐based CAD for lung nodule detection. In this work, we again attempted to learn from the clinical procedures, and tried to identify those aspects that could be mimicked in algorithm design. In particular, for clinical evaluation of a scan, radiologists would commonly take the axial, coronal and sagittal planes into account, rather than solely the axial plane. However, previous work on nodule detection is mostly based on the axial plane alone.^20^, ^23^, ^27^, ^30^ The influence of using orthogonal planes including axial, coronal, and sagittal views for nodule detection in a deep learning‐based CAD system has not been explored. Additionally, radiologists’ sensitivity on small nodules is not high on CT scans in clinical practice.^31^, ^32^, ^33^ The system aims to provide better small nodule detection by combining multiple planes.

The key contributions of this paper are as follows. (a) Considering the axial plane, the coronal plane, and the sagittal plane, we developed an automatic nodule identification system based on multiplanar convolutional neural networks using transfer learning. (b) We also explored the performance and influence of each plane for nodule detection in a CAD system. Combined results from three planes on the detection performance were reported. To further boost the performance, results of the proposed system on 10 mm axial maximum intensity projection‐based slices were merged since the 10 mm slices had the highest detection rate and a relatively low FP rate found in the previous work.^34^ (c) Based on convolutional neural networks, a multiscale dense architecture was applied to exclude suspicious candidates. Features at low or high levels can be extracted and concatenated for prediction. (d) In the FP reduction stage, we evaluated the effect of two factors: Segmentation of lung parenchyma and the region of interests of input data. (e) Although it is difficult to detect small nodules (i.e., nodules with a diameter <6 mm), our designed CAD system achieved good performance on these small nodules.

## 2 MATERIALS AND METHODS

The designed method contained two stages, namely, multiplanar nodule candidate detection and FP reduction. We used a convolutional neural network model, U‐net++, to detect potential nodule candidates on axial, coronal, and sagittal planes. The backbone of the U‐net++ was the Efficient‐Net classification model, pretrained on ImageNet, which extracts various basic features. The predictions from the three planes were merged to acquire a higher sensitivity. For FP exclusion, we applied multiscale dense convolutional neural networks to remove FP candidates. The following sections provided more details of the dataset, architectures, training progress, and evaluation methodology.

### 2.A Dataset

The public dataset named Lung Image Database Consortium and Image Database Resource Initiative (LIDC‐IDRI) was established by seven academic centers and eight medical imaging companies.^35^ The database had 1018 CT scans and the range of the slice thickness is from 0.6 to 5.0 mm. These scans were reviewed by four radiologists in two reading phases. In the first round, radiologists independently detected suspicious lesions and categorized them into three groups (nodules ≥3 mm, nodules <3 mm, and non‐nodules). Then, findings of each scan from four radiologists were collected together and individual radiologist checked every annotation again in an unblinded way.

In clinical practice, scans with low slice thickness are recommended for pulmonary nodule detection.^36^ Hence, we excluded scans with slice thickness above 2.5 mm. After discarding scans without consistent slice spacing, there were 888 scans included in our study. Nodules larger than 3 mm were considered as relevant lesions according to NLST screening protocols.^6^ Since no consensus among four radiologists was provided, suspicious nodules detected by the minority of radiologists could be FPs. Thus, we selected 1186 nodules which were accepted by at least three radiologists as the reference standard. Nodules ≥ 3 mm identified by the minority of radiologists, nodules <3 mm, and non‐nodules were referred as unrelated findings. In the LIDC‐IDRI database, radiologists gave 5 scores (1 = ground‐glass, 2–4 = part‐solid, 5 = solid) for nodule types. If the majority of votes are 1 and 5, the nodule type is ground‐glass and solid, respectively. Otherwise, the nodule type is part‐solid. Consequently, there were 64 ground‐glass, 189 part‐solid, 933 solid nodules in the study. Nodule size also was provided in the database. When the nodules were stratified by size as suggested by the lung CT screening reporting and the data system,^37^ the study had 502 nodules (3–6 mm), 276 nodules (6–8 mm), 281 nodules (8–15 mm), 127 nodules (≥15 mm).

### 2.B Data preparation

**Fig. 1:**
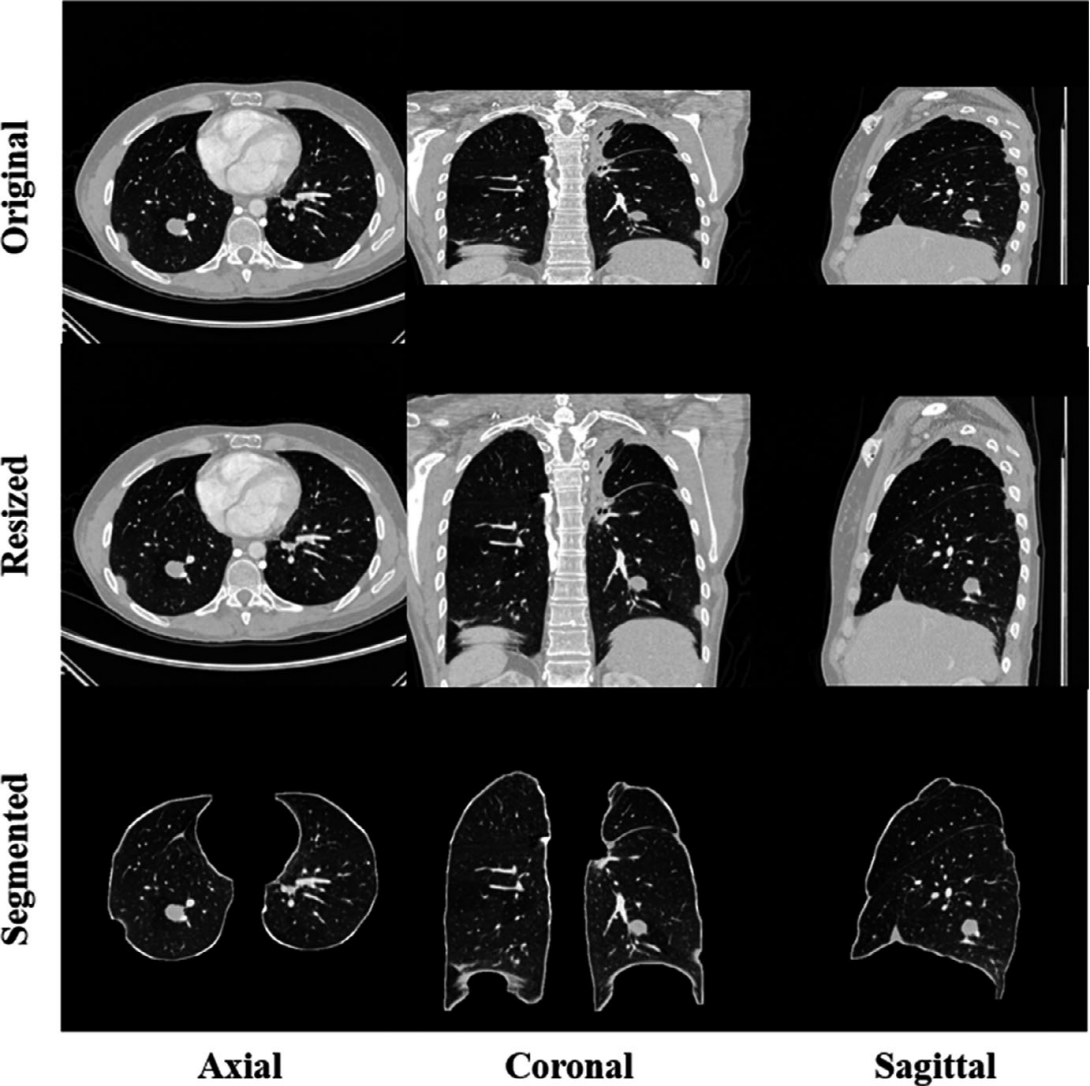
Examples of preprocessing for axial, coronal, and sagittal slices. The first column is original computed tomography (CT) data and the second column represents slices after resizing by interpolation. In the last column, segmented lung parenchyma in various directions is applied as input for training convolutional neural networks later.

We applied the window setting of (−1000 HU, 400 HU) and converted DICOM to images in an 8‐bit PNG format since it is convenient for our developers to make visual comparisons between inputs and outputs (preprocessed images, predictions) of our model during processing. Images were normalized to the range between 0 and 1 during the model training. Scans in the LIDC‐IDRI database have various spacing in different planes, which results in misshapen images for nodule detection. Original examples are shown in the first row of Fig. 1. To unify the data, we adopted 1 mm as the spacing value to resize the images by interpolation since thin‐thickness slices improve diagnosis.^38^ Moreover, segmentation of lung parenchyma can increase efficiency of training convolutional neural networks for lung nodule detection.^39^ The average of pixel values in the whole slice was applied as a threshold to roughly separate lung parenchyma from the body. We removed the irrelevant information in the border and employed morphological closing to fill holes. To keep more boundary texture for detection of wall‐attached nodule, morphological operation, dilation, was used. The segmentation procedure is described and illustrated in more detail in our previous work.^30^ Segmented lung parenchyma in three planes is illustrated in the third row of Fig. 1.

### 2.C Multiplanar detection via transfer learning

Nodule candidate detection is a fundamental step as it is highly related to the final sensitivity of the CAD system. To achieve as high sensitivity as possible, we applied not only axial slices but also coronal and sagittal slices for nodule candidate localization. The reason of utilizing three planes is that one nodule might be not obviously showing in one plane. To further improve detection accuracy, we combined our previous work and used 10 mm axial maximum intensity projection (MIP) based slices generated for nodule detection. The output of the candidate detection stage is constructed by using a union join from the output of four CNNs streams. More specifically, detected candidates on coronal and sagittal plane were first transformed back to axial coordinates. Potential candidates are merged if the largest radius of the candidates is smaller than 0.88 times central distance between the two. A smaller ratio leads to much more FPs and the same number of detected nodules, whereas a larger ratio results in a lower sensitivity at this stage. The final detection performance in both situations will decrease. Hence, the optimal experimental ratio, 0.88, is applied in the study.

**Fig. 2:**
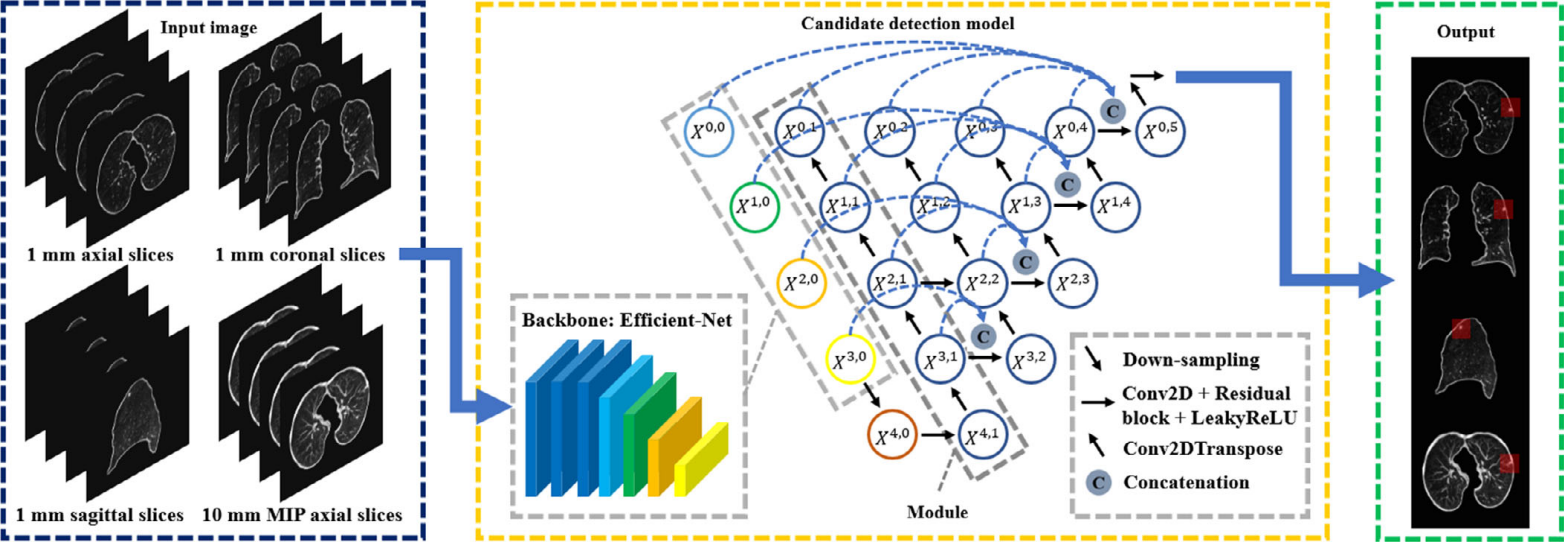
The overview of our proposed candidate detection method. The 1 mm slices on axial, coronal, sagittal plane, and 10 mm axial maximum intensity projection (MIP) slices are used as input. In the encoder part, the backbone of the detection model is based on efficient‐net pretrained on ImageNet. In the decoder part, the node is represented by $X^{i,j}$ where the integer i (0≤i≤4) denotes the transpose two‐dimensional convolutional layer along the decoder and the integer j (1≤j≤5) denotes the convolution layer along the skip pathway. The proposed model extracts features not only in small receptive fields but also large receptive fields. After prediction, suspicious findings on each plane are localized by bounding boxes.

With its a series of dense skip pathways between decoder and encoder networks, U‐net++ shows good performance in segmentation tasks.^15^ Based on U‐net++, we proposed our object detection model, as shown in Fig. 2. Input slices and ground truth images have a size of 512×512. The nodule in the ground truth image is labeled by a square bounding box, with a width set to the nodule diameter provided by the LIDC‐IDRI dataset.

The architecture has two parts, namely encoder and decoder. For the encoder part, we adopt Efficient‐Net^16^ as backbone because it is more efficient on simple feature extraction and had the promising results on the CIFAR‐100 image classification task. The model Efficient‐NetB4 was pretrained on the ImageNet dataset, and its pretrained weights were downloaded from the python package website (https://pypi.org/project/keras‐efficientnets/). Using a pretrained model based on a large dataset such as ImageNet, and then retraining (also called fine‐tuning) that model on a different dataset for a different task is known as transfer learning. Transfer learning has shown good results on different tasks in the past,^40^, ^41^, ^42^ and the main benefits of it include that the model will already have rich feature maps prior to fine‐tuning which can speed up the training and give better performance on other datasets. Efficient‐Net has a compound scaling method, which results in eight versions of the Efficient‐Net. The method applies a compound coefficient μ to constrain width (w), depth (d), and resolution (r) in networks:

(1) $$d=\alpha^{\mu }$$

(2) $$w=\beta^{\mu }$$

(3) $$r=\gamma^{\mu }$$

where α, β, and γ are constants and greater than or equal to 1. To avoid amount of computation more than $2^{\mu }$, the product of $\alpha \cdot \beta^{2}\cdot \gamma^{2}$ is close to two. The width, depth, resolution, and the dropout rate of EfficientNet‐B4 that we use are 1.4, 1.8, 380, and 0.4, respectively, established by a grid search experiment.^16^ The output of the backbone is connected to Leaky Rectified linear units (LeakyReLU) with a negative slope coefficient of 0.1.^43^ Since the learning rate ($10^{‐3}$) at this stage is relatively large, this may cause the dying ReLU problem. Thus, LeakyReLU is used to extend the range of ReLU and prevent vanishing gradients in parts of the network. Then, it is followed by a max‐pooling layer and a dropout layer. The dropout rate in this architecture is 0.1. In the decoder part, the node is represented by $X^{i,j}$ where the integer i (0≤i≤4) denotes the transpose 2D convolutional layer along the decoder and the integer j (1≤j≤5) denotes the convolution layer along the skip pathway. In the middle of the U‐Net++, the first convolutional layer consists of 256 kernels of size 3×3 between $X^{4,0}$ and $X^{4,1}$. In order to increase the depth of the model, we apply two residual blocks which have 256 channels with LeakyReLU as the activation function. The decoding pathway consists of five similar modules. The first module ($X^{4,1}\to X^{3,1}\to X^{2,1}\to X^{1,1}\to X^{0,1}$) is made of four transposed 2D convolutional layers, one concatenation layer, one dropout layer, one convolutional layer, and one residual block with LeakyReLU as the activation function. More specifically, in order to revert the spatial compression, we employ four transpose 2D convolutional layers with 128 kernels of size 3×3 for up‐sampling.^44^ Then, the concatenation layer combines related feature maps from transposed convolutional layers at previous one level and the corresponding layer in the encoding pathway (backbone: Efficient‐NetB4). At each horizontal level, all concatenated feature maps are merged on the ultimate node on that level (nodes $X^{3,2}$, $X^{2,3}$, $X^{1,4}$, $X^{0,5}$). After the concatenation layer followed by one drop‐out layer (drop rate: 0.1) and one convolutional layer with 128 kernels, there is a 128‐channel residual block activated by LeakyReLU. For the following four modules, the number of transposed convolutional layers is reduced by one and the number of channels/kernels is halved for each subsequent module. For example, the second module is comprised by the pathway $X^{3,2}\to X^{2,2}\to X^{1,2}\to X^{0,2}$. The last module is almost the same as the fourth module. However, it does not have the concatenation layer and has one more dropout layer with the rate 0.05. The last layer is a convolutional layer with a kernel size of 1×1 and a sigmoid activation function. After prediction, suspicious findings on each plane are localized by bounding boxes.

**Fig. 3:**
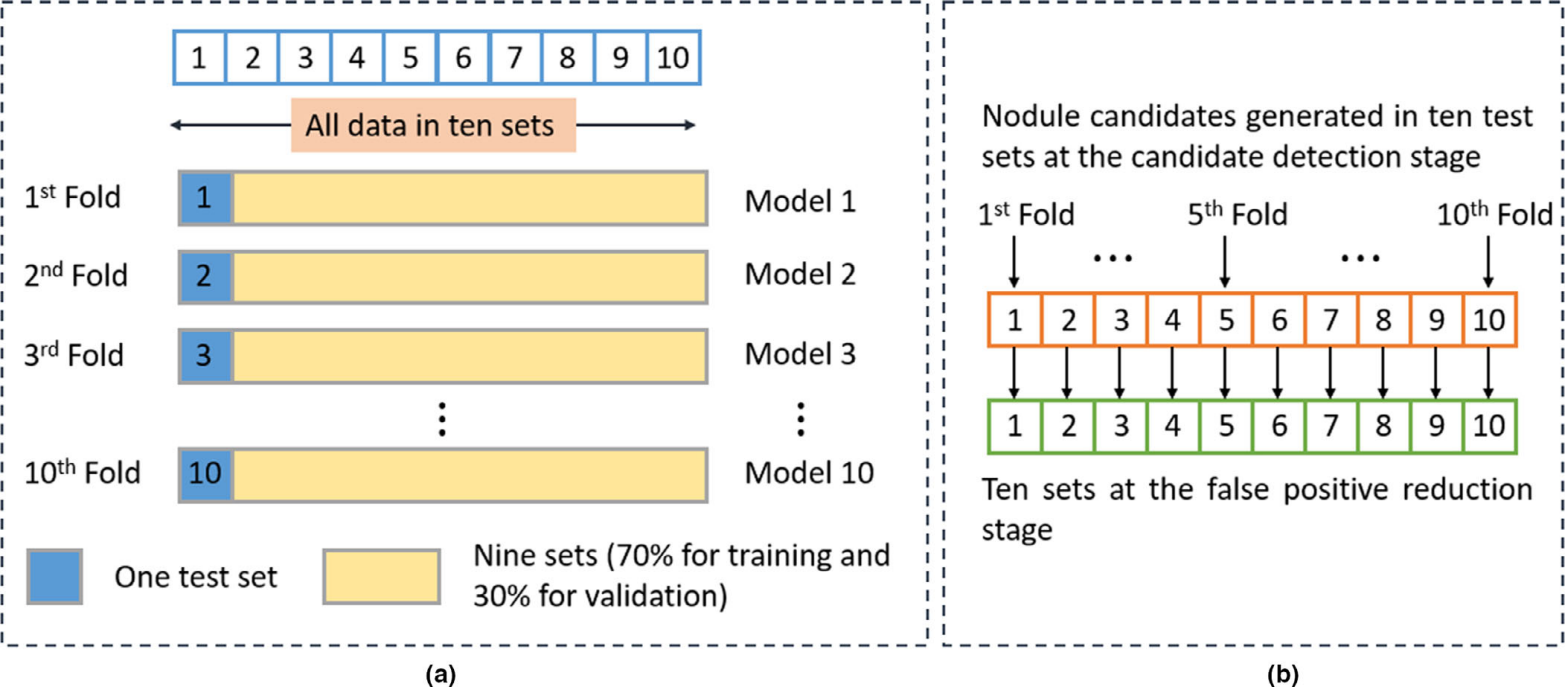
Illustrations of the training scheme. (a) presents the principle of the tenfold cross‐validation for both nodule candidate detection and false positive (FP) reduction. (b) shows how the ten sets at the FP reduction stage are generated.

In the training stage, each input image has at least one nodule. During the training, image augmentation, such as 0°, 90°, 180°, and 270° rotations, horizontal–vertical flipping, and horizontal–vertical shift, is randomly performed on the fly. Images are not rotated by arbitrary degrees since arbitrary rotations might change nodule image characteristics due to interpolation and in turn reduce the detection performance. The data were separated by the LUNA16 challenge into ten sets.^23^ We use tenfold cross‐validation for model development, as shown in Fig. 3(a). Specifically, every fold of the cross‐validation consists of training, validation, and testing data. In each fold, we leave one set of data for testing. The remaining data are split into a training set (70%) and a validation set (30%). The validation set here is used for model evaluation for each of the hyper‐parameter sets during the training. The test repeats ten times until every set is used as the independent testing set once and the detected nodules on each set are then aggregated. We use a batch size of 8 and the Adam optimizer.^45^ To calculate the overlap between ground truth and prediction, we apply dice as the loss function.^46^ The initial learning rate is $10^{‐3}$ and the minimum value is $10^{‐4}$. The decreasing factor of the learning rate scheduler is set to $10^{‐1}$. If the minimum validation loss does not change for 5 epochs, the learning rate decreases. The training ends when the model minimum loss on the validation set does not change for 10 epochs.

### 2.D Multiscale dense training for false positive reduction

**Fig. 4:**
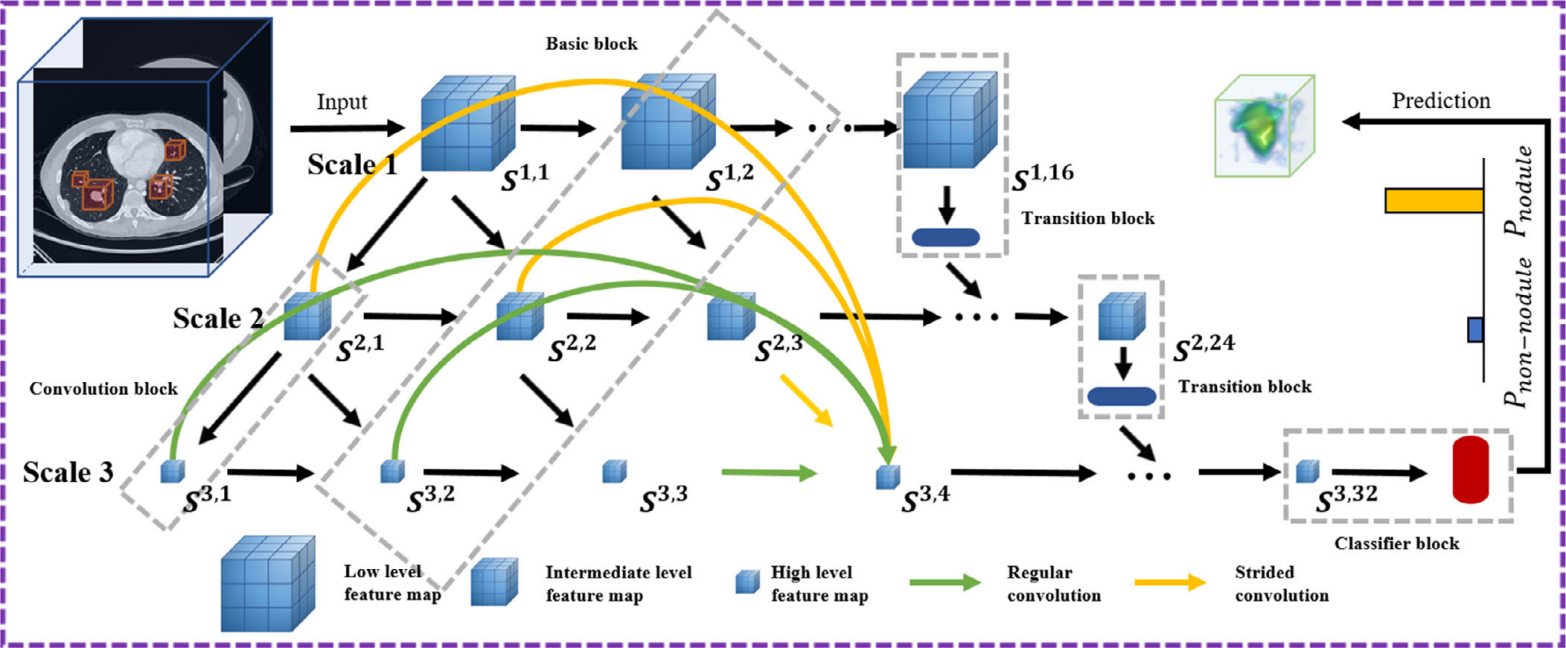
The scheme for false positive reduction based on three‐dimensional (3D) multiscale dense convolutional neural networks. The cubes are extracted from 3D volume as input. Feature maps are in three scales and the scale 1, 2, 3 has a depth of 16, 24, and 32, respectively. An example of concatenated feature maps from different levels through regular and strided convolutions is shown in scale 3 at layer 4. The classifier is in the end of scale 3, giving a probability of being a nodule for each cube. The node is represented by $S^{i,j}$ where the integer i (1≤i≤3) denotes the scale level in the vertical direction and the integer j (1≤j≤32) denotes the depth in the horizontal direction. The $P_{\mathrm{nodule}}$ and $P_{non‐nodule}$ represent the probability of being a nodule and a non‐nodule, respectively. Connection between different scales and horizontal layers is not drawn explicitly.

Reduction of FPs is also essential for radiologists in clinical practice. The aim of this stage is to lower the number of nodule candidates so that fewer nodule candidates have to be manually inspected, ultimately reducing the workload of radiologists. The model that we propose here is based on 3D multiscale dense convolutional neural networks,^18^ as shown in Fig. 4. Overall, the model has feature maps at three different scales and a maximum depth of 32 in the vertical and the horizontal direction, respectively. The node is represented by $S^{i,j}$ where the integer i (1≤i≤3) denotes the scale level and the integer j (1≤j≤32) denotes the depth. Green arrows indicate regular convolution operations in the horizontal path, while orange arrows represent strided convolution operations in the vertical path. Feature maps are extracted and concatenated from the results of regular convolutions on the same scale and the result of strided convolutions on the previous scale. Connection between different scales and horizontal layers are not drawn explicitly. But an example on scale of 3 at a depth of 4 with green and orange arrows is shown in Fig. 4. The network consists of 32 basic blocks, five transition blocks, and a classifier block. The architecture starts with three convolution blocks to extract initial feature maps in three scales. Each convolution block contains a convolutional layer with a kernel size of 3×3×3 followed by batch normalization with the activation function ReLU.^47^ Because a small learning rate is applied to train the model and the chance of having dead neurons is low, ReLU, therefore, can be used for this task. On three scales, their numbers of filters are 32, 64, 128, and growth rates are 8, 16, 32, separately. A basic block includes three concatenation layers and five bottleneck operations that are used to reduce the number of features and improve calculation efficiency. Every bottleneck operation consists of two convolution blocks. After the bottleneck block, the number of filters is reduced by 75%. On scales 2 and 3, coarse and fine features are aggregated by concatenation from the previous and current scales. When extracting features by the strided convolution from the previous scale, the stride depth is two rather than one for a larger receptive filed. To further improve model compactness, transition blocks are designed to reduce the number of feature maps. A transition block has a convolution block with a stride of one and a kernel size of 1×1×1. The transition blocks are connected to the basic block and located at a depth of 16 and 24 in three scales. The final block is a classifier block which gives, for each input cube, the probability of containing a nodule. It has two convolutional blocks, an average pooling layer with stride of two, a flatten layer, two dense layers (128, 32 filters), and two dropout layers with the rates of 0.5 and 0.2 separately. The initializer in the convolutional layer is the he_normal.^48^

Before the training session, each candidate needs to be rescaled to 32×32×32. The rough size of every candidate is first estimated in the candidate detection stage, which gives a bounding box for candidates according to their diameters. However, the surrounding textural information also influences the differentiation between nodules and non‐nodules for convolutional neural networks. Therefore, we experiment with two parameters that govern the availability of textural context to the FP reduction model: (a) Whether or not the lung parenchyma is segmented and (b) size of the region of interests of the input data.

Figure 3(b) describes how the ten sets for FP reduction are generated. At the nodule candidate detection stage, in every fold, nodule candidates, including FPs and true positives, are generated by a nodule detection model. As a consequence, ten sets are created at the nodule candidate detection stage. These ten sets are used directly as the ten sets for FP reduction. The tenfold cross‐validation scheme is then applied to train, validate, and test the FP reduction models, as shown in Fig. 3(a). After tenfold cross‐validation, the performance on the full set is calculated. The loss function is binary cross‐entropy and the optimizer is Adam. The learning rate is $10^{‐4}$. If the validation loss does not change for 6 epochs, the training stops.

### 2.E Performance evaluation

At the nodule candidate detection stage, sizes and types of detected nodules from our proposed CAD system are analyzed. In our case, the number of true positives is much smaller than the number of FP findings. Using the area under the receiver operating characteristic (ROC) curve as an evaluation metric therefore does not reflect the performance of the CAD system objectively.^49^ Thus, we used the Competition Performance Metric (CPM),^50^ which calculates the average sensitivity at seven FP rates (1/8, 1/4, 1/2, 1, 2, 4, and 8 FPs/scan) in the free‐response ROC (FROC) curve for assessment.^49^ After tenfold cross‐validation, the predictions for all ten test sets were combined to compute the performance and 95% confidence intervals on the full dataset, using bootstrapping with 1000 bootstraps.^51^

The proposed scheme is implemented by applying deep learning library of Keras based on a graphics processing unit (GPU), NVIDIA V100.^52^

## 3 RESULTS

### 3.A Nodule candidate localization

The performance of the system at nodule candidate detection stage on each plane, as well as the fused results are presented in Table I. The sensitivity acquired by 1 mm axial slices, 1 mm coronal slices, and 1 mm sagittal slices is 91.1%, 82.5%, and 81.8%, respectively. Details of the contributions of each stream and nodules only identified by the coronal or sagittal stream are in the supplementary material. When the results of 1 mm axial and coronal slices are combined, the sensitivity improves from 91.1% to 94.9% as same as the sensitivity after fusing the results of 1 mm axial and sagittal slices. When we merge the results from 1 mm axial, coronal and sagittal slices, the system achieves a sensitivity of 96.1%. Upon combining the results from the 10 mm axial MIP images, the CAD system detects 98.1% of lung nodules. This proves that every stream provides complementary information for nodule candidate localization, especially the axial plane. Normally, a high sensitivity implies many FPs from the CAD system. With 1 mm axial slices, 1 mm coronal slices, 1 mm sagittal slices, and 10 mm axial MIP images, our proposed method has 38, 33, 40, and 22 FPs/scan, respectively. The FP rate is 98 FPs/scan after fusing results from three 1 mm planes, whereas the number of FPs/scan is 108 by fusion of candidates from four streams.

**Table I:** Performance of the CAD program using 1 mm slices in three directions and 10 mm axial maximum intensity projection (MIP) images as input, as well as fused results at the nodule candidate detection. Total number of nodules is 1186 within 888 scans.

Input data	Number of detected nodules	Sensitivity (%)	False positives per scan
1 mm axial slices	1081	91.1%	38
1 mm coronal slices	979	82.5%	33
1 mm sagittal slices	970	81.8%	40
10 mm MIP images	1105	93.2%	22
Fusion 1 mm slices	1140	96.1%	98
Fusion all	1163	98.1%	108

The summary of detected lung nodules in size and density type according to the Lung CT screening reporting and the data system at the nodule candidate detection stage is shown in Table II. The main missed nodules are in the small‐size group (3–6 mm), there are three ground‐glass nodules and 12 solid nodules undetected. However, the detection rate of small nodules is still 97.0%. Regarding nodules larger than 6 mm, only eight nodules are missed and the detection rate is 98.8%. For ground‐glass, part‐solid, and solid nodules, the detection rate is 90.6%, 100%, and 98.2%, respectively.

**Table II:** Performance when combining results of multiple planes at the nodule candidate detection stage.

Nodule diameter	Nodule type			Total
	Ground‐glass	Part‐solid	Solid	
3–6 mm	25 (89%)	75 (100%)	387 (97%)	487 (97%)
6–8 mm	13 (93%)	41 (100%)	220 (100%)	274 (99%)
8–15 mm	18 (95%)	48 (100%)	211 (99%)	277 (99%)
≥15 mm	2 (67%)	25 (100%)	98 (99%)	125 (98%)
Total	58 (91%)	189 (100%)	916 (98%)	1163 (98%)

### 3.B False positive candidate exclusion

**Fig. 5:**
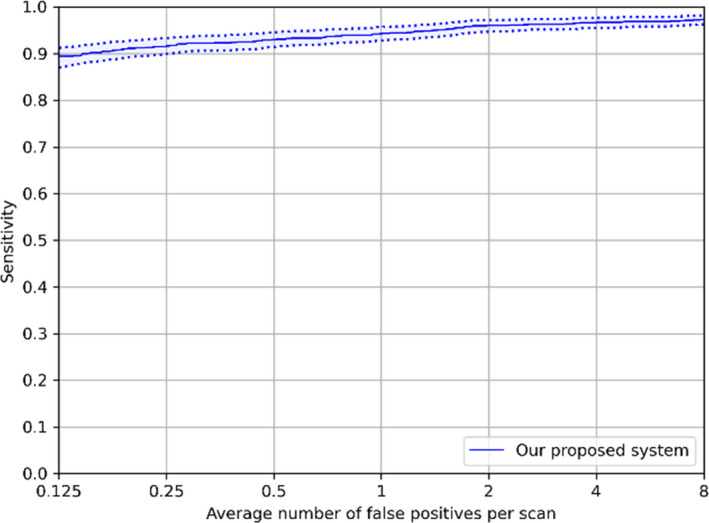
Free‐response receiver operating characteristic (FROC) curves of our proposed system in different configurations. The performance was computed based on 95% confidence interval using bootstrapping with 1000 bootstraps.

Our developed system in these configurations is assessed by FROC curves, as shown in Fig. 5. The system has a sensitivity of 94.2% with 1.0 FP/scan and 96.0% with 2.0 FPs/scan regardless of nodule size. For detection of nodules smaller than 6 mm, the designed CAD system detects 93.4% (95.0%) of these small nodules at an overall FP rate of 1.0 (2.0) FP/scan. The CPM score of the CAD scheme with varied configurations at the FP reduction stage is shown in Table III. Applying 1 mm unsegmented axial slices in the size with four extra pixels has the best CPM score (0.9403) that is slightly higher than the score when the patch size with eight extra pixels is used. Compared to that, using 1 mm.

**Table III:** Performance of the CAD scheme with varied configurations at the false positive reduction stage.

Segmentation	Region of interest	CPM
Segmented	Original	0.9326
Unsegmented	Original	0.9371
Unsegmented	Four pixels larger	0.9403
Unsegmented	Eight pixels larger	0.9401

segmented axial slices acquires a lower CPM score (0.9326). Hence, we adopt the 1 mm unsegmented region of interest with four extra pixels on the axial plane as input data for FP exclusion. The experimental results show that the remaining lung boundaries can slightly improve the final performance of nodule identification. Examples of true positive nodules, false negatives and FPs after FP reduction are shown in Fig. 6.

**Fig. 6:**
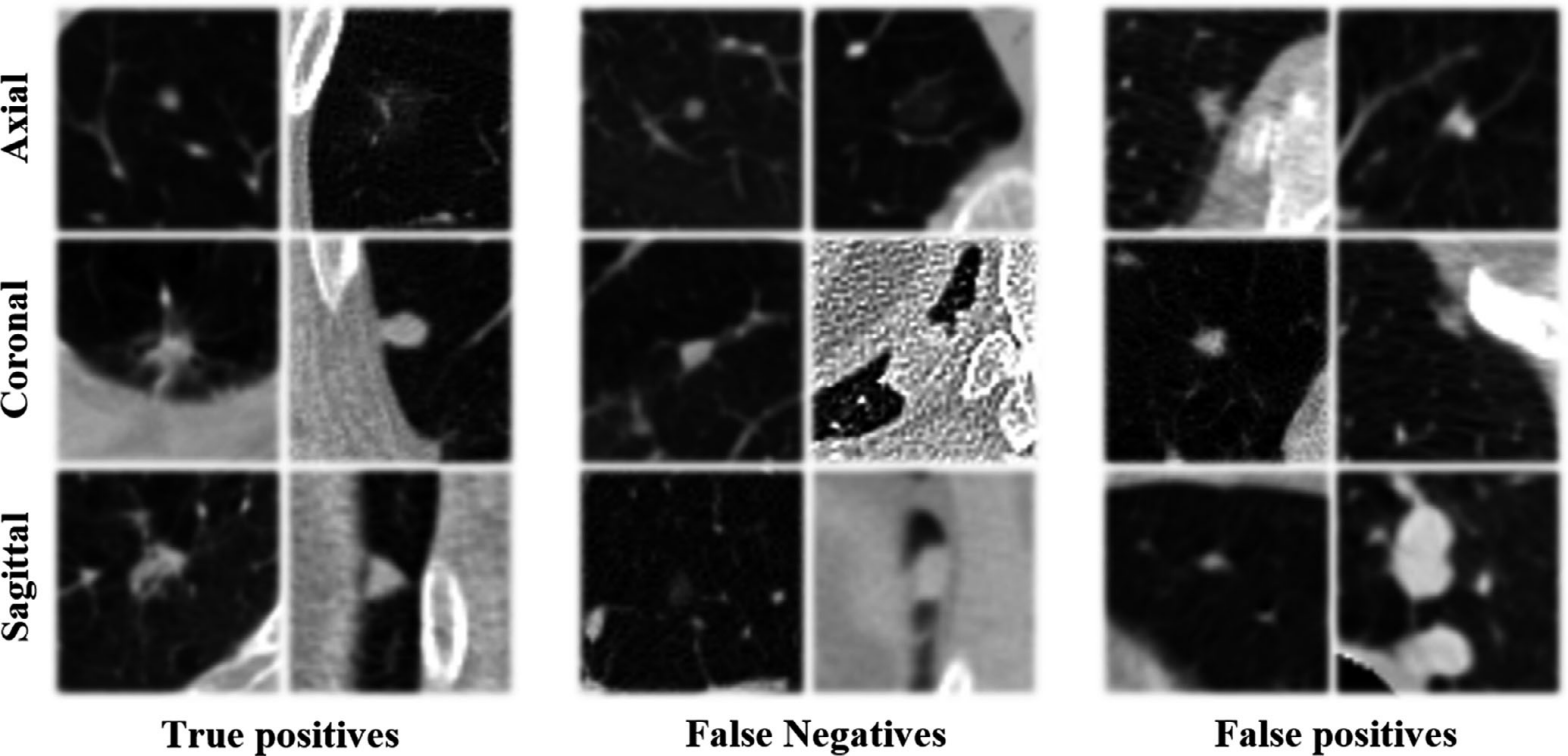
Examples of true positive nodules, false negative ones, and false positives.

### 3.C Comparison with published nodule detection systems

To benchmark the performance of our complete CAD program, we list the results from other published papers which were obtained on the same dataset. Sensitivities at different FP rates in other methods are shown in Table IV.

**Table IV:** Performance of other computer‐aided detection systems evaluated on the LIDC‐IDRI database.

CAD system	Year	False positives/scan							CPM
		0.125	0.25	0.5	1	2	4	8	
Our current work	2020	**0.893**	0.917	0.930	0.942	0.960	0.966	0.973	0.940
Setio et al.^23^	2017	0.859	**0.937**	**0.958**	**0.969**	**0.976**	**0.982**	**0.982**	0.952
Zhang et al.^27^	2018	0.890	0.931	0.944	0.949	0.965	0.972	0.976	0.947
Zheng et al.^30^	2019	0.876	0.899	0.912	0.927	0.942	0.948	0.953	0.922
Ozdemir et al.^24^	2019	0.832	0.879	0.920	0.942	0.951	0.959	0.964	0.921
Wang et al.^20^	2019	0.788	0.847	0.895	0.934	0.952	0.959	0.963	0.905
Huang et al.^26^	2019	0.817	0.851	0.869	0.883	0.891	0.907	0.914	0.876
Dou et al.^21^	2017	0.677	0.737	0.815	0.848	0.879	0.907	0.922	0.826
Xie et al.^22^	2019	0.734	0.744	0.763	0.796	0.824	0.832	0.834	0.790

The highest score of each column is shown in bold.

## 4 DISCUSSION

We proposed a novel lung nodule detection system based on multiple planes using convolutional neural networks. The aim of this study was to improve the performance of the deep learning‐based CAD system for automatic pulmonary nodule detection. Our method achieved comparable performance among the CAD systems evaluated on the LIDC‐IDRI database. The combined results from three planes showed better performance than the result from any individual single plane, indicating different planes can provide complementary information for lung nodule detection.

Nodule detection performance was evaluated on the axial, sagittal, and coronal planes separately. The axial plane outperforms the rest of two planes in 1 mm slice thickness, achieving a detection sensitivity of 91.1%. A possible reason is the difference in image quality. Since nearly 90% of scans have a slice thickness larger than one millimeter, the image resolution in coronal and sagittal planes is low. After interpolation and scaling, image noise might increase and the morphological information of nodules in coronal and sagittal planes might be lost, especially for small nodules. In contrast, the resolution (pixel spacing small than one) in axial slices is higher. This keeps more nodule information for convolutional neural networks to better differentiate nodules from other anatomical structures. In a study regarding human reader performance, it was found that radiologists also have a higher sensitivity, but more FPs for nodule detection on the axial plane compared to the coronal one.^53^ In clinical practice, the sagittal plane might be the last option for radiologists to find nodules since vessels tend to be presented as cross sections in this direction. The section of vessels can result in more suspicious findings during reviewing. Through experiments, our study found most of the FP candidates on the sagittal plane. When we fused the results from three 1 mm planes and 10 mm axial MIP images, the sensitivity increased from the lowest sensitivity of 81.8% to 98.1%, although the number of FPs increased. This suggests that incorporating multiple planes can be an effective approach for 2D nodule detection. At the FP reduction stage, we also found that leaving the lung parenchyma unsegmented and using a larger region of interest of extra four pixels in radius boosts the performance of classification. This implies that CNNs can be more accurate to differentiate nodules and FP findings with more surrounding information.

In a recent study it was shown that detection of small nodules (i.e., nodules with a diameter <6 mm) is the main challenge for which the sensitivity of CAD systems is difficult to improve.^20^, ^23^, ^27^ We analyzed detected nodules at the candidate localization stage. Our method had a sensitivity of 97% on detection of these small nodules in various types. There are only 15 of 502 lung nodules still missed by our method. The detection rate of these small nodules is high since with the help of skip connections, U‐net++ can efficiently extract features not only in small receptive fields but also large receptive fields. Interestingly, there is no missed part‐solid nodule. The reason might be that unlike solid nodules, having nonsolid components helps part‐solid ones to be easier differentiated from section of vessels. Moreover, their morphology is more distinguishable compared to ground‐glass nodules for convolutional neural networks. Note that the proposed method found 99% of nodules (>6 mm) in large morphological variations. However, there are still some missed nodules. These nodules may appear in unusual locations or close to tissues, which makes detection more difficult for the system.

Recent published approaches on the LUNA16 challenge were summarized in Table IV. To compare the results using the same criteria, we only listed the methods which used the competition performance metric (CPM) with sensitivities at seven FP rates. Our designed method was ranked third and had the highest sensitivity when the number of FPs allowed is equal to 0.125 FP/scan. The top 1 is from the work of Setio et al.^23^. With gaining benefits from different CAD systems, they have a better sensitivity when more FPs are allowed. The CPM score from Zhang et al.^27^ is also higher and detecting all possible nodule candidates gives them a good upper‐bound quality for the FP exclusion stage. The work by Ozdemir et al.,^24^ Wang et al.,^20^ and our previous study demonstrates that a high sensitivity can be achieved,^30^ but the large number of FPs per scan that are generated incurs extra reading time for radiologists. The CAD system we propose here shows good performance in detecting these small nodules even after the FP reduction stage, representing a higher sensitivity than radiologists’.^31^, ^32^, ^33^ We also improve our performance in detection of nodules smaller than 6 mm, compared to our previous work (sensitivity: 93.4% vs 90.4%, at 1.0 FP/scan; sensitivity: 95.0% vs 91.6%, at 2.0 FPs/scan).^30^ Another study from Ozdemir et al.^24^ showed a sensitivity of around 90% with 1FP/scan for nodules smaller than 5 mm, whereas our method achieved a sensitivity of 93.0% with 1FP/scan to detect nodules with the same size. Methods of Dou et al.,^21^ Xie et al.,^22^ and Huang et al.^26^ might need to further improve the discrimination between nodules and wrong findings. Some systems did not report sensitivities at various FP rates. For instance, Cui et al. developed a nodule detection system using 39 014 scans from multiple centers.^29^ Although the system reached a sensitivity of 93.4% with 0.8 FP/scan, a number of true nodules were still missed by the system when the FP rate was smaller than 0.5. Nevertheless, the external validation results showed the potential use of the deep learning‐based system in clinical practice. Additionally, Gruetzemacher et al.^25^ utilized a 3D U‐net with more spatial information for detection, which results in a high sensitivity of 92.7% but 4 FPs/scan. The system also might have an issue of extra screening time for radiologists due to a large FP rate. Besides, with the help of shape‐based features and texture‐based knowledge, Tan et al.^28^ achieved good results on nodule detection using part of the dataset. However, the performance on the whole dataset was unknown.

The study has several limitations. Firstly, the reference standard is not derived by the consensus of radiologists but consists of nodules accepted by at least three radiologists. The system might find some true nodules only identified by one or two readers and the performance of the system can be underestimated. Secondly, the dataset has imbalanced numbers of nodules in size and density for system development. For example, the number of solid nodules is 14 times the number of ground‐glass ones. Thus, the system tends to detect suspicious solid nodules and might miss ground‐glass nodules. This may affect the performance of the system when it is tested on other datasets. Last but least, the system is developed and evaluated based on the scans that might from the same vendors or protocols via a cross‐validation scheme, which might lead to a positive bias. There are some suggestions for the future work. Although this developed CAD system had good performance on this large public dataset, evaluations on lung cancer screening programs need to be carried out. Another interesting topic is that with larger memory in GPUs, convolutional neural networks are capable to be trained by the whole 3D lung volume for nodule detection. The system might achieve better performance since vessels and pulmonary nodules can be easily differentiated in 3D space.

## 5 CONCLUSIONS

We have developed a multiplanar nodule detection system using convolutional neural networks. The promising performance has shown the effectiveness of combining results from three planes for the candidate detection task. Sharing multiscale features helped dense convolutional neural networks to become more effective for removal of FPs.

## CONFLICT OF INTEREST

Matthijs Oudkerk is the chief scientific officer at the institute named i‐DNA. Sunyi Zheng is partly employed by the institute i‐DNA as an AI advisor. Ludo J. Cornelissen is partly employed by the company named COSMONiO for medical AI research. The other authors have no relevant conflict of interest to disclose.

## Supporting information


**Fig. S1**. Examples of nodules which are only identified on one plane. (a) The nodule is only detected on the coronal plane. (b) The nodule is only found on the sagittal plane.

**Table S1**. Performance of using 1 mm axial slices in the detection of nodules at the candidate detection stage.

**Table S2**. Performance of using 1 mm coronal slices in the detection of nodules at the candidate detection stage.

**Table S3**. Performance of using 1 mm sagittal slices in the detection of nodules at the candidate detection stage.

**Table S4**. Performance of using 10 mm MIP slices in the detection of nodules at the candidate detection stage.

**Table S5**. Performance when combining results on 1 mm sagittal, 1 mm axial and 10 mm MIP slices in the detection of nodules at the candidate detection stage.

**Table S6**. Performance when combining results on 1 mm coronal, 1 mm axial and 10 mm MIP slices in the detection of nodules at the candidate detection stage.

**Table S7**. Performance when combining results on 1 mm axial and 10 mm MIP slices in the detection of nodules at the candidate detection stage.

**Data S1**. Contributions of each plane in the candidate generation process.
