# Supplementary figures and images for: Deep convolutional neural networks for multiplanar lung nodule detection: Improvement in small nodule identification
Source: Med Phys. 2020 Dec 30;48(2):733–44. doi: 10.1002/mp.14648 (PMC7986069; doi:10.1002/mp.14648)

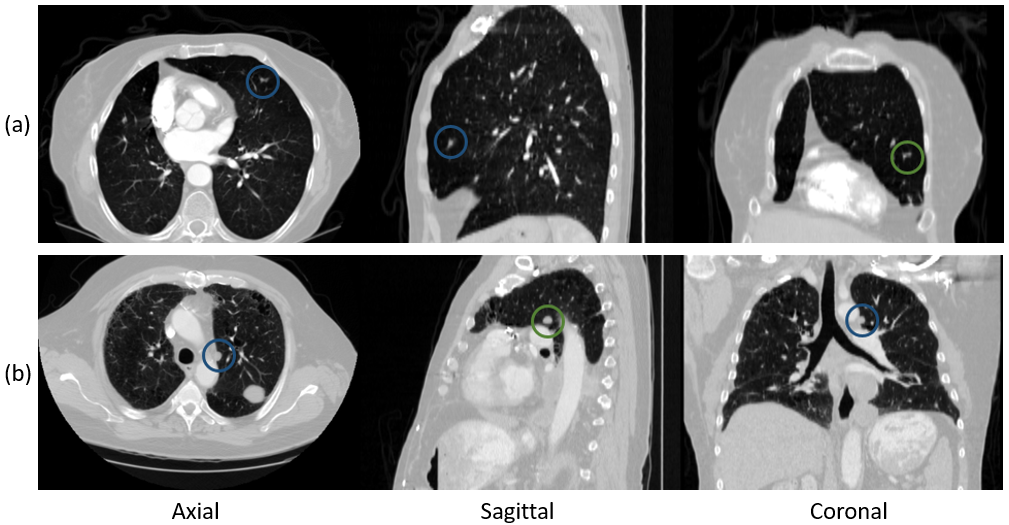

Supplement: Supplementary file 1 — Fig. S1. Examples of nodules which are only identified on one plane. (a) The nodule is only detected on the coronal plane. (b) The nodule is only found on the sagittal plane. [file MP-48-733-s009.tif]
