# Supplementary material for: Deep convolutional neural networks for multiplanar lung nodule detection: Improvement in small nodule identification
Source: Med Phys. 2020 Dec 30;48(2):733–44. doi: 10.1002/mp.14648 (PMC7986069; doi:10.1002/mp.14648)
Supplement: Supplementary file 2 — Table S1. Performance of using 1 mm axial slices in the detection of nodules at the candidate detection stage. [file MP-48-733-s007.docx]

**Table S-1.** Performance of using 1 mm axial slices in the detection of nodules at the candidate detection stage.

| Nodule diameter | Nodule type | | | Total |
| --- | --- | --- | --- | --- |
|  | Ground-glass | Part-solid | Solid |  |
| 3-6 mm | 23 | 69 | 346 | 438 |
| 6-8 mm | 11 | 41 | 205 | 257 |
| 8-15 mm | 17 | 46 | 200 | 263 |
| \| $\geq$15 mm \| \| --- \| | 2 | 25 | 96 | 123 |
| Total | 53 | 181 | 847 | 1081 |
