# Supplementary material for: Deep convolutional neural networks for multiplanar lung nodule detection: Improvement in small nodule identification
Source: Med Phys. 2020 Dec 30;48(2):733–44. doi: 10.1002/mp.14648 (PMC7986069; doi:10.1002/mp.14648)
Supplement: Supplementary file 5 — Table S4. Performance of using 10 mm MIP slices in the detection of nodules at the candidate detection stage. [file MP-48-733-s004.docx]

**Table S-4.** Performance of using 10 mm MIP slices in the detection of nodules at the candidate detection stage.

| Nodule diameter | Nodule type | | | Total |
| --- | --- | --- | --- | --- |
|  | Ground-glass | Part-solid | Solid |  |
| 3-6 mm | 20 | 70 | 366 | 456 |
| 6-8 mm | 9 | 41 | 215 | 265 |
| 8-15 mm | 15 | 46 | 202 | 263 |
| \| $\geq$15 mm \| \| --- \| | 2 | 24 | 95 | 121 |
| Total | 46 | 181 | 878 | 1105 |
