# Supplementary material for: Deep convolutional neural networks for multiplanar lung nodule detection: Improvement in small nodule identification
Source: Med Phys. 2020 Dec 30;48(2):733–44. doi: 10.1002/mp.14648 (PMC7986069; doi:10.1002/mp.14648)
Supplement: Supplementary file 8 — Table S7. Performance when combining results on 1 mm axial and 10 mm MIP slices in the detection of nodules at the candidate detection stage. [file MP-48-733-s001.docx]

**Table S-7.** Performance when combining results on 1 mm axial and 10 mm MIP slices in the detection of nodules at the candidate detection stage.

| Nodule diameter | Nodule type | | | Total |
| --- | --- | --- | --- | --- |
|  | Ground-glass | Part-solid | Solid |  |
| 3-6 mm | 24 | 72 | 376 | 472 |
| 6-8 mm | 13 | 41 | 218 | 272 |
| 8-15 mm | 17 | 47 | 207 | 271 |
| \| $\geq$15 mm \| \| --- \| | 2 | 25 | 98 | 125 |
| Total | 56 | 185 | 899 | 1140 |
