# Supplementary material for: Deep convolutional neural networks for multiplanar lung nodule detection: Improvement in small nodule identification
Source: Med Phys. 2020 Dec 30;48(2):733–44. doi: 10.1002/mp.14648 (PMC7986069; doi:10.1002/mp.14648)
Supplement: Supplementary file 9 — Data S1. Contributions of each plane in the candidate generation process. [file MP-48-733-s008.doc]

Deep convolutional neural networks for multi-planar lung nodule detection: improvement in small nodule identification

**SUPPLEMENTS**

From the tables above, we can find that the stream of using 1 mm axial slices detects more nodules in all different sizes and types (Table S-1), compared to the streams that apply 1 mm coronal and sagittal slices (Table S-2 and S-3). In addition, there is no large difference between the streams that use 1 mm coronal and sagittal slices except 10 solid nodules smaller than 6 mm. The stream with 10 mm axial slices finds the most nodules and is good at detecting solid nodules compared to other streams (Table S-4). To analyze the extra contributions of different streams, we take the results of 1 mm axial slices as the baseline since they are the images regularly used by radiologists and other CAD methods. When the results of 1 mm axial and coronal slices are combined, the sensitivity improves from 91.1% to 94.9%. It has the same sensitivity when the results of 1 mm axial and sagittal slices are merged. However, the sensitivity (96.1%) is higher when the results of 1 mm axial, coronal and sagittal slices are fused. This shows that 1mm slices in every direction can contribute to the detection of nodules. Moreover, the sensitivity is 96.1% after combining results of 1 axial slices and 10 mm MIP slices, whereas the sensitivity is 98.1% when the results of four streams are merged. This also indicates the potential benefits of taking 1 mm coronal and sagittal slices into consideration.

Furthermore, we have analyzed the nodules that are only found by the sagittal or coronal stream. In Table S-5, it shows the combined results on 1 mm sagittal, 1 mm axial and 10 mm MIP slices. Six solid nodules are detected by the coronal stream only (Table II and S-5). Five out of six have a diameter between 3 and 6 mm and the remaining one has a diameter between 6 and 8 mm. In order to analyze nodules only found by the sagittal stream, results on 1 mm coronal, 1 mm axial and 10 mm MIP slices are merged, as shown in Table S-6. Similarly, six nodules, including one ground-glass nodule, one part-solid nodule, and four solid nodules, are localized by the sagittal stream only (Table II and S-6). Regarding the diameter of these six detected nodules, 50% of nodules are in diameter between 3 and 6 mm and the other three nodules have a diameter between 8 and 15 mm. Some examples of nodules only identified on one plane are shown in Figure S-1. Besides, twenty-three more nodules are detected by using coronal or sagittal planes (Table II and S-7). Among these twenty-three nodules, eleven of them are detected on both two planes. They are one ground-glass nodule, three part-solid nodules, and seven solid nodules. Seven out of eleven nodules are smaller than 6 mm. The aforementioned results suggest that taking the coronal and sagittal planes into account not only increases the detection rate of different types of nodules (ground-glass, part-solid or solid), but also yields an improvement in identifying nodules smaller than 15 mm.

**Figure:**

**Figure S-1.** Examples of nodules which are only identified on one plane. (a) The nodule is only detected on the coronal plane. (b) The nodule is only found on the sagittal plane.
